# Springing or Warping of Plates

**Published:** 1853-07

**Authors:** S. D. Muse

**Affiliations:** Sharon, Miss.


### *Springing or Warping of Plates.* By S. D. Muse, D. D. S. of Sharon, Miss.

Dr. Taylor:—As much has been written of late on the subject of "springing or warping of plates in soldering," and as most operators seem to have a full share of trouble on account of such accidents, (if we may so term it,) and I in common have borne a part in the common difficulty, and as all seem disposed to throw all possible light on the subject both as to cause and remedy, and being disposed to contribute my mite, (for know ye that I also have an opinion—a theory as to cause and remedy.) Although I may be considered by my professional brethren to have fallen as far short, as to the true cause and the remedy, as any of them, ye- you may take it for what it is worth, but test it before you set your estimate.

Some attribute the misfortune to impurity of plate, to too much copper; others to over much solder; others, again, to beating up the work irregularly, over-heating, etc., etc. And as these opinions are entertained by gentlemen of acknowledged scientific ability, men who have been in regular practice, may be for fifteen or twenty years, it would almost savor of presumption in me who am a practitioner of five or six years, (you must know I had not the advantage of a course in a Dental College until the winter of '46 and '47, when I attended the Ohio College of Dental Surgery, in which you were and are a revered professor,) to set up a theory of my own, discarding theirs in toto. However, I am bound to do so, in view of all the facts.

I have tried the remedies—they have failed; then the cause cannot be that, or those ascribed. As to remedies, it is needless to attempt to enumerate them all. A few, however, may not be out of place. The most plausible we give.

One says, use as little alloy in both plate and solder as possible. This will prevent the plate from drawing up, or shrinking, which results from consumption of the baser metals.

Says another, use cast iron boxes, or rings to enclose the work, to hold the plaster plate and teeth to their place.

Another advises that two or three teeth be attached at a time so that the plate may not be too generally heated at once. More of them might be given if necessary.

As to the first, we contend that it is erroneous, because we frequently see very good fitting plates, which are not more than 16 caret, with 12 carret solder, the latter very abundant at that.

The second will not succeed owing to the greater expansibility of the iron ring, than the enclosed plaster-sand and plate. The ring will generally be found loose before the job is ready for the blow-pipe, hence it (the ring) does not confine the work, and is of little use, if any.

The third is still farther from the mark, in my judgment, for had I a wish to spring the plate, I should as readily accomplish the object by heating one point to redness, while the remainder is left comparatively cool, as by any other method.

I would not be thought to speak lightly of any gentleman or gentlemen, who have endeavored to throw light on this perplexing subject; on the contrary, I feel grateful to all, for their kind efforts to impart useful knowledge on this, as all other professional matters. The spirit it manifests is in beautiful contrast with the conduct of those who happening on an idea, (or borrowing one,) either lock it up in their own selfish pate, or put the stop law of patent on it, that we may not use it without their permission, which, of course must be paid for very liberally. With the latter I have no sympathy whatever, and I am of opinion that they should forthwith return all they have borrowed, and close themselves, tarrapin like, in their own shells, and claim no fraternal relations with an honorable, liberal, and scientific profession.

I will now endeavor to explain my theory of cause or causes, which is, or are very simple, namely: 1st. Heating up the job too suddenly, thereby expanding the outer portion of the mass, before the central portion has received a like amount of heat, and equal expansion, thereby drawing the plate out of shape.

And by the difficulty of keeeping the ring and plaster mass at the same temperature after being placed under the blow pipe, the heat, being mainly directed to the teeth, and the metalic ring (let it be copper or iron, being a better conductor of caloric than the mass,) now contracts on the mass, and springs the plate out of shape.

The third and last is, the want of regular temperature throughout the operation, (I mean the keeping the mass of uniform temperature.)

I am aware that it is essential to have the work enclosed so as to prevent the mass in which the plate and teeth are embedded, from crumbling off, which is all the purpose the ring can subserve, which it frequently does to the destruction of the operation.

Then if this all be true, and I have a better method, the profession ought to be put in possession of it. As I am a large borrower, and should pay back a moiety, if possible, more for the remedy, or remedies.

Take a piece of iron wire about (for the want of a guage plate, and not recollecting size from No.,) the size of a small rye straw, or half a line in diameter, bend it to the following form: ΛΛΛΛ. Prepare enough in this way to form a belt to enclose the piece, and put it in shape by bringing the two ends together, and fastening them. It will be necessary to have the belt when complete nearly or quite as deep as the common iron ring, box or band, as you may please to term it.

Another form will answer about as well thus, |_|‾|_ I prefer the first, however, as I think it has more elasticity or flexibility. Having completed your belt, take a piece of wire the same size, and bend so as to encircle the belt about $\frac{1}{4}$ of an inch all round, now take very small wrapping wire, and tie the wire to the belt, at opposite points, so as to extend it a little all round, being careful to draw it as much at one point as at another. About half a dozen ties will be sufficient. Now you are ready to place your paper or pasteboard rim round your belt, when this is done, and you have poured in your paste of sand and plaster, and imbeded the job therein all having set, take off the paper rim and clip the ties which bind the outside wire to the belt, and your job is ready for the fire. I never attempt to raise the heat on a piece until it is thoroughly dried. Then I do it in the following manner, which is one of the important secrets of success, in my opinion.

I take the best charcoal I can get, place some fire on the hearth, then build up in the form of a cone, until I have a sufficient quantity to heat up the job, which I now place on the top, having put on solder, etc. In this way the work heats up evenly and gradually. I now (seeing the mass becoming pretty warm,) begin to place burning coals around, keeping one all the time over the teeth. When the mass is at a lively red heat I place it on a piece of charcoal scooped out, and previously laid on the fire to heat up, and in short order all is done, and the plate has not sprung.

This wire belt has several advantages over the ring. 1st. It has a constant tendency to contract without the power to crush the mass, or derange the work. 2d. It will, in proportion as the plate expands, yield to or from; it does not come loose around the work, and lastly, is not so cumbersome.

